# Poncet's disease: An uncommon presentation of a common disease in Sudan

**DOI:** 10.1002/ccr3.8032

**Published:** 2023-10-10

**Authors:** Abdalla Mohamed Abdalla Mohamedali, Ahmed Mahmoud Sayed Sayedahmed, Fatima Abdelmoneim Ahmed Elmustafa, Abdelrahman Abdelgader Mohammed Alkhair Ebrahim, Mahmoud Saeed Saad Mahgoub, Nihad Abdelghayoum Abbass Mohamedkheir, Ahmed Mohamed Abdalla Mohamed Ali

**Affiliations:** ^1^ Trauma & Orthopedics Department Omdurman Islamic University Omdurman Sudan; ^2^ Omdurman Islamic University Omdueman Sudan; ^3^ University of Khartoum Khartoum Sudan; ^4^ Cairo University Giza Egypt

**Keywords:** general medicine, immunology, infectious disease, rheumatology

## Abstract

**Key Clinical Message:**

Poncet's disease is an acute onset reactive polyarthritis associated with tuberculosis infection. Although uncommon, the diagnosis should be considered among patients presenting with symmetrical polyarthritis in tuberculosis‐endemic regions.

**Abstract:**

This is a case report of Poncet's disease presenting as bilateral knee and wrist pain associated with swelling. Joint x‐rays and immunological assays were normal. A chest x‐ray and Gene‐Xpert diagnosed tuberculosis. A complete resolution of symptoms was attained following the completion of antituberculous therapy regimen.

## Graphical abstract

Graphical Abstract text: This chest x‐ray shows right‐sided hilar lymphadenopathy and apical consolidation, which are typical features of tuberculosis. The atypical aspect of this case report is that Poncet's disease (tubercular rheumatism) was the initial presentation of tuberculosis.

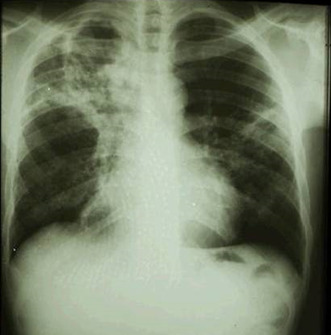

## 1 INTRODUCTION

Sudan is one of the developing countries in which tuberculosis (TB) is considered as a major health concern. In 2019, the World Health Organization estimated that the number of new cases of TB in Sudan was 29,000.^1^

TB is a multisystemic disease that has the ability to affect any organ. The skeletal system is involved in 1%–3% of TB cases, and the spine is the most commonly involved skeletal organ.^2^

Poncet's disease (also known as Tubercular rheumatism) is a rare form of TB which usually manifest as symmetrical polyarthritis without evidence of joint invasion by Mycobacterium TB.^3^ Given the rarity of Poncet's disease, the diagnosis is usually considered when all other causes of such a presentation (symmetrical polyarthritis) have been excluded.^4^ We present a case report of Poncet's disease as an initial presentation of TB in an undiagnosed patient in Sudan.

## 2 CASE PRESENTATION

A 38‐year‐old female presented to the referral clinic with bilateral knee and wrist pain and swelling for 1 month. In addition, she reported pain in the metacarpophalangeal joints of both hands with associated morning stiffness. On further questioning, she reported cough, fever, and night sweating for 3 months. According to Sudanese Vaccination Protocol, she had a complete immunization status, including the BCG vaccine. Family history of TB, autoimmune diseases, or rheumatologic diseases was denied.

Her vital signs were: a temperature of 38.4°C, a respiratory rate of 19 cycles per minute, a pulse rate of 98 beats per minute, and an SpO_2_ of 94%. On examination, she was found to be pale. The rheumatologic examination revealed swelling and tenderness in both knees, both wrists, and the metacarpophalangeal joints of both hands. Apart from mild crepitations over the right upper lobe, the chest examination was normal. To add, further clinical examination as unremarkable. The complete blood count (CBC) showed mild microcytic hypochromic anemia with raised erythrocyte sedimentation rate (ESR) (85 mm) and C‐reactive protein (CRP) (12 mg/L). The autoimmune workup was negative (antinuclear antibody and rheumatoid factor). X‐rays of her affected joints were normal (no evidence of erosion).

**FIGURE 1:**
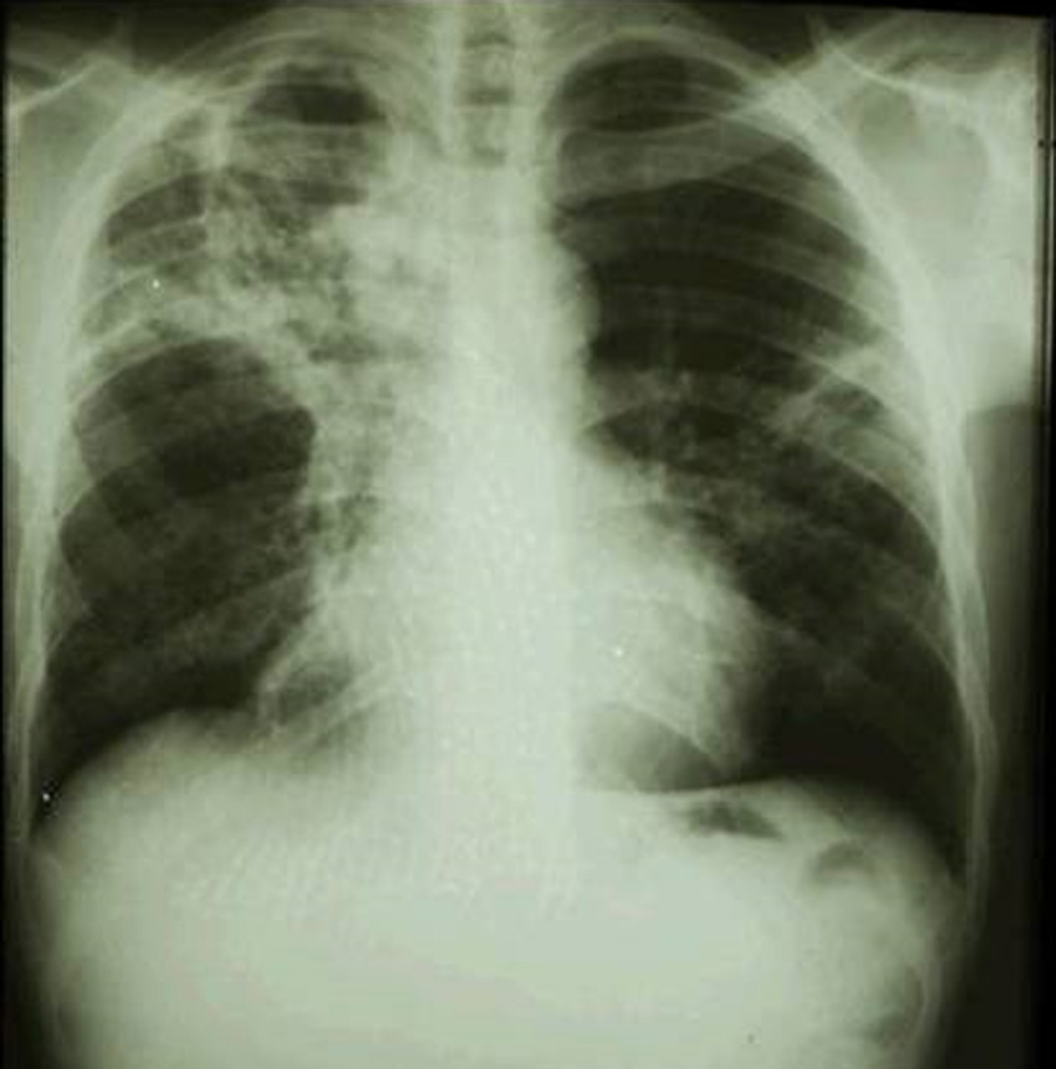
Chest X‐ray. The chest x‐ray shows right sided hilar lymphadenopathy in addition to apical consolidation.

Owing to her clinical picture and the prevalence of TB in Sudan, chest x‐ray (CXR) and Gene‐Xpert test of sputum were performed. The CXR showed right sided hilar lymphadenopathy as well as apical consolidation (Figure 1), and the Gene‐Xpert of sputum detected mycobacterium TB.

Based on that, the antituberculous therapy was started using the following drug regimen: rifampicin, isoniazid, ethambutol, and pyrazinamide. The four medications were used for 2 months, following this period, the ESR and CRP returned to normal ranges. In addition, rheumatologic symptoms were completely resolved. Following this, the rifampicin and isoniazid were continued for 4 months. Adherence to medication was emphasized and monitored during the management period. Six months following the completion of the antituberculous therapy, she was in good health and showed complete resolution of symptoms.

## 3 DISCUSSION

In this study, the patient presented with bilateral knee, wrist, and metacarpophalangeal joint pain in addition to morning stiffness. Although the presentation is typical for systemic rheumatological diseases, the autoimmune work‐up was negative, while the Gene‐Xpert was positive for TB. Lourenço et al reported a similar presentation.^5^ In their case report, the patient experienced asymmetrical oligoarthritis (knees and ankles) in addition to erythema nodosum but these were not supported by the typical immunological markers.^5^ This, accompanied by a positive interferon gamma release assay test pointed towards the diagnosis of TB and subsequently Poncet's disease (PD).^5^

In an additional study conducted by Garg et al. among 18 patients with acute inflammatory ankle arthritis, eight patients were diagnosed with PD based on a positive Mantoux test and CT scan of the chest showing mediastinal and/or paratracheal and/or unilateral hilar lymphadenopathy with central necrosis.^6^ The accuracy of the results are questioned by the lack of microbiological and histopathological confirmation however it showcase the importance of including PD as a differential diagnosis of acute ankle arthritis in TB‐endemic regions.^3^

Polyarthritis is the presenting feature in our case, consistent with findings of the literature.^5^ Knees and ankles were the most commonly involved joints followed by the wrists in a review by Ktroot et al.^5^ while Knees followed by small joints of the hands particularly MCP joints were the most commonly involved joints in a case series by Abdulaziz et al.^3^

An additional feature of Poncet's disease found in this study is the absence of joint erosion in the x‐rays of the affected joints, as Poncet's is classically defined as an arthritis that develops in the acute onset of TB and resolves following the commencement of anti TB medications, without causing joint destruction.^5^, ^6^ This finding was also reported by Lourenço et al and Higashiguchi et al.^5^, ^7^

A review of the literature showcases the historical association of PD with extrapulmonary TB.^8^ However, from the description of 198 PD cases in the literature, the site of TB infection was demonstrated in 96.5% with 56.8% being extrapulmonary in nature.^6^ Another review of 52 cases of PD reported an extrapulmonary site only in 48% of cases.^5^ While an extended review of the literature from 2007 to 2012 reported the finding of an extrapulmonary infection in cases of PD to be 60%,^3^ and exceeding 70% in their own cohort of patients.^3^

In this case, the arthritis resolved after 6 weeks of the commencement of antituberculous therapy. This finding is comparable to another study which concluded that the clinical presentation of PD was short lived and the arthritis subsided in an average of 51.6 days following the initiation of therapy.^6^

## 4 CONCLUSION

This case‐report sheds the light on an uncommon manifestation of TB. Given this, in regions endemic with TB, the diagnosis of Poncet's disease should be considered among patients presenting with arthritis where more common causes are excluded.

## AUTHOR CONTRIBUTIONS

**Abdalla Mohamedali:** Conceptualization; project administration; supervision; validation; writing – original draft; writing – review and editing. **Ahmed Sayedahmed:** Conceptualization; validation; visualization; writing – original draft; writing – review and editing. **Fatima Elmustafa:** Validation; visualization; writing – original draft; writing – review and editing. **Abdelrahman Mohammed Alkhair:** Visualization; writing – original draft; writing – review and editing. **Mahmoud Mahgoub:** Validation; writing – original draft; writing – review and editing. **Nihad Mohamedkheir:** Validation; writing – original draft; writing – review and editing. **Ahmed Ali:** Validation; writing – original draft; writing – review and editing.

## FUNDING INFORMATION

This case report did not receive any form of funding.

## CONFLICT OF INTEREST STATEMENT

Authors declare no conflict of interest

## ETHICS STATEMENT

Patient confidentiality was preserved in this report and all ethical considerations were done in accordance with the Declaration of Helsinki.

## CONSENT

A written informed consent was obtained from the patient prior to the writing and submission of this case report.

## Data Availability

Data sharing not applicable to this article as no datasets were generated or analysed during the current study.
